# Advances in Kidney Preservation Techniques and Their Application in Clinical Practice

**DOI:** 10.1097/TP.0000000000003679

**Published:** 2021-03-18

**Authors:** Sarah A. Hosgood, Rachel J. Brown, Michael L. Nicholson

**Affiliations:** 1 Department of Surgery, University of Cambridge, Addenbrooke’s Hospital, Cambridge, United Kingdom.

## Abstract

The use of cold preservation solutions to rapidly flush and cool the kidney followed by static cold storage in ice has been the standard kidney preservation technique for the last 50 y. Nonetheless, changing donor demographics that include organs from extended criteria donors and donation after circulatory death donors have led to the adoption of more diverse techniques of preservation. Comparison of hypothermic machine perfusion and static cold storage techniques for deceased donor kidneys has long been debated and is still contested by some. The recent modification of hypothermic machine perfusion techniques with the addition of oxygen or perfusion at subnormothermic or near-normothermic temperatures are promising strategies that are emerging in clinical practice. In addition, the use of normothermic regional perfusion to resuscitate abdominal organs of donation after circulatory death donors in situ before cold flushing is also increasingly being utilized. This review provides a synopsis of the different types of preservation techniques including their mechanistic effects and the outcome of their application in clinical practice for different types of donor kidney.

## Graphical abstract

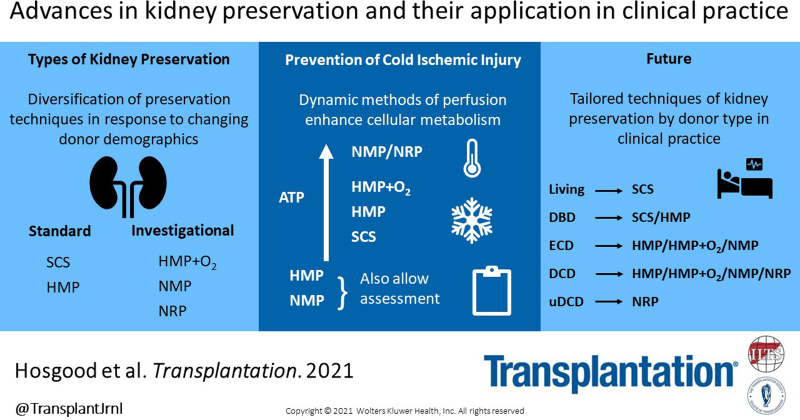

## INTRODUCTION

Techniques of kidney preservation traditionally rely on the principle of hypothermia. Hypothermic temperatures inhibit enzymatic processes and reduce metabolism by 2- to 3-fold for every 10°C reduction in temperature.^1^ This slows the depletion of ATP and also inhibits the degrading processes. Nonetheless, over time, the gradual depletion of energy substrates leads to a loss of cell viability and the development of necrosis.^1,2^ The cold ischemic time (CIT) alone is an independent risk factor for graft failure and is directly associated with delayed graft function (DGF).^3^ The risk of DGF increases by 23% for every 6 h of cold ischemia (CI) and if the CIT is limited to <12 h, the risk of DGF is reduced by 15%.^4^ The CIT also significantly increases allograft immunogenicity, provoking acute and chronic rejection.^5,6^

In the last decade, driven by rising transplant waiting lists, more extended criteria donor (ECD) and donation after circulatory death (DCD) donor kidneys are considered for transplantation. Older donor organs have higher immunogenicity and increased risk of graft dysfunction and failure.^5,6^ The use of uncontrolled DCD (uDCD) donors is also increasing, with firmly established programs in several European countries.^7,8^ ECD, controlled DCD (cDCD), and uDCD kidneys are all particularly susceptible to CI.^9-11^ Efforts are made to limit the preservation interval where possible. Nonetheless, geographical and logistical constraints often result in more prolonged durations of CI, particularly in the United States. Different modes of mechanical preservation using modified hypothermic and normothermic techniques are being investigated to minimize or reverse the effects of CI and some of these have found their way into clinical practice.

This review summarizes the outcomes of kidney transplantation from different types of organ donor, then details the different kidney preservation techniques, exploring their mechanistic actions and application in clinical practice stratified by organ donor type.

## KIDNEY DONORS

### Live Donor Kidney Transplantation

Living related or unrelated kidney donor transplantation has excellent outcomes. Rates of DGF, graft, and patient survival are detailed in Table 1.^12-15^ Kidneys have a short period of warm ischemia (WI; 4–10 min) under normal circumstances followed by a brief period of CI (mean, 2–6 h). Nonetheless, kidney exchange programs and donation have increased over the last few years and without the capacity to transfer patients in some centers, CIT can be much longer. Evidence from the United States, Australia, the United Kingdom, and Europe has demonstrated that shipping kidneys is safe with CITs of up to 16 h. However, this increases the risk of DGF, which may reduce graft and patient survival and increase incidences of acute rejection.^12,34-37^

**TABLE 1.** Outcomes of kidney transplantation from different donor types (% range)

Donor type	DGF rate (%)	Graft survival at 1 y (%)	Graft survival at 5 y (%)	Patient survival at 1 y (%)	Patient survival at 5 y (%)	References
Living	2–19	96–98	85–92	98–99	93–95	^12-15^
DBD	10–28	89–97	80–85	96–99	82–89	^16-19^
ECD	28–38	82–92	49–80	90–96	70–93	^4,9-11,20^
DCD	23–58	85–92	77–87	95–99	82–89	^7,16-19,21-26^
uDCD	42–93	85–100	60–87	83–98	78–94	^8,23,27-33,38^

DBD, donation after brain death; DCD, donation after circulatory death; DGF, delayed graft function; ECD, extended criteria donor; uDCD, uncontrolled DCD

### Deceased Donor Kidney Transplantation

Rates of DGF in deceased donor kidney transplantation range from 10% in standard donation after brain death (DBD) kidneys to 93% in uDCD^4,7,8,10,16-23,27,38^ (Table 1). The outcomes of graft and patient survival at 1 y are good but slightly lower in comparison to living donor kidneys.^8-11,18,20,21,24-33,38^ At 5 y, there is a marked reduction in graft and patient survival, in particular in ECD kidney transplants (Table 1).^8-11,18,20,21,24-33,38^

## KIDNEY PRESERVATION TECHNIQUES

### Static Cold Storage

Static cold storage (SCS) is the simplest form of organ preservation. Organs are flushed with cold preservation solution at approximately 4 °C, then stored in solution on ice until transplanted. Preservation solutions are designed to allow adequate flushing of the microcirculation and rapidly cool the kidney. They contain a number of critical components to maintain cellular viability during storage at 4 °C: an impermeant to counteract swelling and to provide stability of the cellular ultrastructure, a buffer to prevent the buildup of intracellular acidosis and a balanced electrolyte composition with either a high or low Na^+^/K^+^ ratio, again to counteract cellular swelling. Solutions with a high K^+^ concentration are classified as intracellular and those with a high Na+ as extracellular solutions (Table 2). There are a number of different SCS preservation solutions available for kidney preservation.

**TABLE 2.** Components commonly used in organ preservation solutions

Impermeants	Glucose, lactobionate, mannitol, raffinose, and sucrose
Colloid	HES and PEG
Buffers	Citrate, histidine and phosphate
Electrolytes	Calcium, chloride, magnesium, magnesium sulfate, potassium, and sodium
Antioxidants	Allopurinol, glutathione, mannitol, and tryptophan
Additives	Adenosine, glutamic acid, and ketoglutarate

HES, hydroxyethyl starch; PEG, polyethylene glycol.

Collins solution was one of the first acellular preservation solutions used in clinical transplantation in 1969.^39^ It was later modified and renamed Euro-Collins solution.^40^

Hyperosmolar citrate (HOC), more commonly known as Soltran or Marshall’s solution, was first developed in the 1970s.^41^ HOC uses citrate as a buffer to prevent accumulation of calcium within the cell (Table 2). Its hypertonicity is also designed to prevent fluid entry into cells. It is a relatively inexpensive, nonviscose intracellular solution.

University of Wisconsin (UW) solution developed by Belzer and Southard in the 1980s contains a vast array of ingredients including antioxidants (glutathione) to scavenge oxygen-free radicals, allopurinol to block the activity of xanthine oxidase, and adenosine, an ATP precursor.^42,43^ It is an intracellular solution and also contains the colloid hydroxyethyl starch to prevent cellular swelling (Table 2). It is considered the “gold standard” preservation solution.

Histidine-tryptophan-ketoglutarate (HTK) solution was originally developed as a cardioplegic solution but because of its low viscosity was quickly adopted for abdominal organs.^44^ It is an extracellular solution and uses the impermeant mannitol and histidine as a buffer. It also contains 2 amino acids: tryptophan, to stabilize cellular membranes and prevent oxidant damage; and ketoglutarate, a substrate to support anaerobic metabolism (Table 2).

Celsior is an extracellular solution and was initially designed for heart transplantation. It contains histidine as a buffer, lactobionate and mannitol to prevent edema, and glutathione as an antioxidant.^45^

Other less well-known solutions include Institut George Lopex-1 solution (IGL-1) and Solution de Conservation des Organes et des Tissus (SCOT, organ and tissue preservation solution).^46,47^ They are extracellular solutions containing polyethylene glycol as a colloid. Hypertonic citrate adenine solution containing citrate and adenine,^48^ phosphate-buffered sucrose 140, developed in the United Kingdom^49^, and Perfudex^50^ and Polysol^51^ are less common solutions. The composition of the more common preservation solutions are listed in several other reviews.^52-54^

#### Live Donor Kidneys

SCS is the main method of preservation for live donor (LD) kidney transplants. There is little evidence for the use of one preservation solution over another in LD kidney transplantation (Table 3). Lynch et al^55^ reported in a series of 475 LD kidneys the rate of DGF was significantly reduced in kidneys preserved in HTK versus UW (3.2% versus 8.2%, *P* = 0.001). UW, HOC, HTK, or Celsior solutions are the most commonly used. In some centers where the CITs are very short, kidneys are simply flushed with a cold crystalloid solution.^77^

**TABLE 3.** Meta-analyses, systematic reviews, randomized controlled trials, case series, and registry studies comparing different preservation techniques in each type of kidney donor

Method of preservation	Donor type	Outcome	References
SCS vs SCS	LD	Case series: HTK reduced DGF vs UW	^55^
	DBD	Systematic review: Euro-Collins increased the risk of DGF. UW equivalent risk of DGF vs HTK and Celsior	^56^
		Registry data: IGL-1 reduced the risk of DGF	^57^
		Randomized study: Celsior improved graft survival vs UW	^58^
	ECD	Randomized study: UW equivalent rates of DGF vs Celsior	^59^
		Registry data: Lower DGF with SCOT solution vs IGL-1	^35^
		Registry data: HTK increased the risk of graft loss vs UW	^60^
	DCD	Meta-analysis: Solutions equivalent	^56^
HMP vs SCS	LD	Case series: Reduced vascular resistance	^61^
	DBD	Systematic review and RCT: Reduced incidences and risk of DGF	^62,63^
		Systematic review: Improved graft survival at 1 + 3 y	^64^
	ECD	Meta-analysis: Reduced the risk of DGF and improved 1-y graft survival	^65^
		RCT: Reduced the risk of DGF, PNF, and improved 1 + 3-y graft survival	^66^
		Registry data: Reduced DGF and improved 1-y graft survival	^67^
		Retrospective study: Equivalent rates of DGF	^68^
	DCD	Meta-analysis: Reduced DGF and improved graft survival at 1 + 3 y	^69^
		Meta-analysis: Reduced DGF	^62^
		RCT: Reduced DGF	^70^
		RCT: Equivalent rates of DGF	^71,72^
		Registry data: Reduced DGF	^73^
Oxygenated HMP vs HMP	DCD	RCT: No significant difference in eGFR at 12 mo. Reduced the incidences of severe complications and acute rejection	^74^
NMP vs SCS			
	ECD	Case series: Reduced DGF	^75^
NRP vs standard in situ cooling	uDCD	Registry study: Reduced PNF and graft loss	^76^

DBD, donation after brain death; DCD, donation after circulatory death; DGF, delayed graft function; ECD, extended criteria donor; eGFR, estimated glomerular filtration rate; HMP, hypothermic machine perfusion; HTK, histidine-tryptophan-ketoglutarate; IGL-1, Institut George Lopex-1 solution; LD, living donor; NMP, normothermic machine perfusion; NRP, normothermic regional perfusion; PNF, primary nonfunction; RCT, randomized controlled trial; SCOT, Solution de Conservation des Organes et Tissus; SCS, static cold storage; uDCD, uncontrolled DCD; UW, University of Wisconsin solution.

#### DBD Kidneys

The main method of kidney preservation is SCS for standard criteria DBD donor kidneys. The most commonly used solutions for SCS include UW, HTK, HOC, and Celsior solutions. Several countries have their own particular preference of solution, for example, IGL-1 is used widely in France but not in other countries.^57^

Evaluation of the best solution for deceased donor kidneys was the subject of much clinical research from the 1970s to early 2000s (Table 3). O’Callaghan et al^56^ conducted a systematic review of 15 trials (3584 kidney transplants) comparing different preservation solutions in all types of deceased donor kidneys. These included 10 randomized controlled trials (RCTs) and 5 non-RCT trials. The authors concluded that the type of preservation solution used influenced the risk of DGF. However, it was not possible to calculate the relative risk (RR) of DGF with any individual solution. Euro-Collins solution was associated with a higher risk of DGF than UW and HTK solutions. UW solution was found to have an equivalent risk of DGF to HTK in 2 RCTs and Celsior in 3 RCTs.^56^ One study found no increased risk of DGF with UW or Euro-Collins solutions with extended CITs.^78^ In agreement, several studies have found similar outcomes with extended CITs between HTK and UW solutions.^55,79^ In the past, HTK solution has been associated with an increased risk of graft loss particularly if the CIT exceeded 24 h.^40,80^

Most recently, Legeai et al^57^ reviewed the outcome of 5 different SCS solutions used for DBD kidneys in France between 2010 and 2014 (n = 7649). The majority (45.5%) were preserved using IGL-1 solution, 9.6% SCOT solution, 29% Celsior solution, 9.6% UW solution, and 5.9% HTK solution. The risk of DGF was significantly lower with the use of IGL-1 solution.

The impact of different preservation solutions on graft survival appears to be minimal, with only 1 study demonstrating improved graft survival with Celsior solution compared to UW solution.^58^

#### ECD Kidneys

There are few well-conducted studies examining the effects of different SCS preservation solutions in ECD kidneys (Table 3). In a single multicenter randomized study including a total of 50 kidneys, Montalti et al^59^ found no difference in rates of DGF when comparing UW (52%) and Celsior solutions (48%). The majority of evidence comes from registry data. Legeai et al^57^ found that the association with DGF and SCOT solutions versus IGL-1 was lower in ECD kidneys (odds ratio [OR] 2.72, 95% confidence interval [CI] 1.98-3.75) compared to standard DBD.^57^ HTK and UW performed similarly irrespective of the CIT. Stewart et al^60^ found using the UNOS registry database that the risk of death-censored graft loss increased when using HTK solution compared with UW solution for ECD kidneys.

#### cDCD Kidneys

SCS is becoming less common for DCD kidneys. However, in the United Kingdom, the majority of cDCD kidneys undergo SCS with UW solution. There are very few studies examining the effects of different preservation solutions for SCS in DCD kidneys (Table 3). In an early series from Japan (2004), Euro-Collins solution was used for SCS of 108 cDCD kidneys. Ninety percent of the kidneys were transplanted but the DGF rate was 86%. The death-censored graft survival was 80% at 5 y and 62.9% at 10 y.^81^

Historically, concerns with the use of HTK solution have been raised regarding its use for DCD kidneys. Some clinical studies have associated its use with the increased risk of primary nonfunction (PNF) and early graft loss. However, meta-analysis including all donor types found no evidence of this in DCD kidneys.^56^

#### uDCD Kidneys

In a UK series of 112 DCD kidneys, the majority uDCD (Maastricht category II donors), Barlow et al^81^ compared the results to 164 matched DBD kidney transplants during the same era (1992–2003). In the DCD series, kidneys were rapidly cooled in situ followed by SCS with HOC solution at 4 °C. The rate of DGF was 83.9% versus 22% (*P* < 0.001) and the PNF rate was 5.4%, similar to the DBD kidneys (1.8%, *P* = 0.164). There was no significant difference in graft survival between the groups up to 15 y posttransplant.

### Hypothermic Machine Perfusion

Hypothermic machine perfusion (HMP) involves the continuous recirculation of cold preservation solution through the kidney at a low pressure. The temperature is maintained between 2 and 8 °C. HMP allows a continual flush of the microcirculation, preventing the accumulation of toxic metabolites. UW solution modified for machine perfusion (MP), UW-MP, is used in most cases for HMP. It was developed by Belzer in the 1960s and provides a source of nutrients and metabolites to support a low level of cellular metabolism to reduce the formation of lactic acid within the cell.^82^ The addition of a colloid (hydroxyethyl starch) is a crucial ingredient to prevent cellular edema during perfusion. The mechanical passage of fluid through the microcirculation also protects against depolarization of the endothelial cell membrane and reduces free radical formation.^83^ Studies have also shown that HMP can increase the release of nitric oxide and reduce endothelin-1, a potent vasoconstrictor produced by vascular endothelial cells.^61,84^ HMP can also reduce levels of inflammatory cytokines^62,85^ and increase phosphorylation of the Akt-Erk signaling pathway to increase the expression of anti-apoptotic and decrease the expression of proapoptotic genes.^86^

#### Assessment

HMP also allows an assessment of viability, which includes the measurement of flow and resistance parameters, analyses of a range of biomarkers in the circulating perfusate and imaging technologies to examine structure. These viability assessments are listed in Table 4 and have been reviewed extensively in several recent publications.^87,88^ The most commonly reported are glutathione S-transferase, lactate dehydrogenase, fatty acid-binding protein, interleukin 18, and measures of lipid peroxidation. Although useful in determining a level of injury, their ability to determine outcome following transplantation is limited.^87,88^

**TABLE 4.** Perfusion characteristics, biomarkers, and imaging techniques used for kidney assessment during HMP and NMP

	HMP	NMP
Perfusion characteristics		
	Flow	Flow
	Intrarenal resistance	Intrarenal resistance
		Macroscopic appearance
Biomarkers		
	GST (tGST, αGST, pi-GST)	Urine production
	LDH	Creatinine clearance
	Lactate	Lactate
	AST	Potassium concentration
	Matrix metalloproteinase 9 + 2	Acid-based homeostasis
	Alanine-aminopeptidase	ET-1
	FABP (H-FABP, L-FABP)	IL-6, IL-8, IL-18
	NGAL	FMN
	Redox-active iron	Lactate
	Lipid peroxidation	AST
	Ionized calcium	Nanoparticle release
	KIM-1	FMN
	FMN	Proteomics
	IL-18	Glucose oxidation
	MicroRNA 21	Transcriptional analysis
	H-NMR	
	Histone H3	
Imaging		
	MRI	MRI
		Contrast-enhanced ultrasound

αGST, alpha GST; AST, aspartate transaminase; ET-1, endothelin-1; FABP, fatty acid–binding protein; FMN, flavin mononucleotide; GST, glutathione S-transferase; HMP, hypothermic machine perfusion; H-NMR, proton nuclear magnetic resonance; IL, interleukin; KIM-1, kidney injury molecule-1; LDH, lactate dehydrogenase; NGAL, neutrophil gelatinase–associated lipocalin; NMP, normothermic machine perfusion; tGST: total GST.

#### Technologies

In the last 2 decades, there has been growing support for HMP in kidney transplantation. There are a number of commercially available portable HMP systems such as the LifePort Kidney Transporter (Organ Recovery System), Kidney Assist (Organ Assist, Groningen, the Netherlands), and WAVES machine (Institut Georges Lopez, Lissieu, France). The RM3 system (Waters Medical Systems, Birmingham, AL) is not portable but is still widely used in the United States.

#### Live Donor Kidneys

There is only 1 report of using HMP in LD kidney transplantation (Table 3). Moser et al^61^ analyzed a series of 16 LD kidneys that underwent HMP and compared them to 16 kidneys that had SCS. The study was powered to detect a difference in vascular resistance measured by Doppler ultrasound. After laparoscopic retrieval, HMP kidneys were flushed with HTK solution and placed on the LifePort Kidney Transporter and perfused with kidney perfusion solution 1. High levels of injury markers were detected in the perfusate (neutrophil gelatinase lipocalin and lactate dehydrogenase) and compared similarly to levels measured in deceased donor kidneys. There were no incidences of DGF or acute rejection in the SCS or HMP kidneys. Resistive indices measured by Doppler ultrasound were significantly less for HMP kidneys postoperatively. The authors concluded that there was a sufficient amount of injury in LD kidneys to warrant the wider use of HMP, particularly in cases where kidneys are shipped and have more prolonged CITs.

#### DBD Kidneys

HMP is becoming more common practice for DBD kidneys in some countries. There have been a number of studies comparing HMP to SCS for DBD kidneys (Table 3). Peng et al^62^ recently performed a systematic review and meta-analysis of RCTs comparing HMP with SCS. A total of 13 RCTs were included in the study. In a subgroup analysis of DBD kidneys, HMP reduced the incidence of DGF compared to SCS (RR 0.78, 95% CI 0.67-0.92, *P* = 0.003) but there was no difference in graft survival at 1 y (RR 1.04, 95% CI 0.99-1.09, *P* = 0.10). Tingle et al^64^ conducted a recent meta-analysis including 971 patients from 4 studies. HMP was found to reduce the risk of DGF in DBD kidneys (RR = 0.77, 95% CI 0.67-0.90, *P* = 0.006). HMP also had a graft survival benefit at 1 and 3 y posttransplant.

Moers et al^63^ reported the largest RCT comparing HMP and SCS from Europe including 672 kidneys from 336 deceased donors. The majority of the kidneys included in the study were from DBD standard criteria donors (SCDs; 78%). They found that HMP reduced the risk of DGF compared to SCS (OR 0.57, *P* = 0.01).

In a further analysis of the European RCT, Kox et al^89^ found that kidneys with short CITs also benefited from HMP, with low rates of DGF. They recommended that HMP should be used for all deceased donor kidneys regardless of the duration of CI. Some centers advocate the use of HMP for more prolonged CI periods to prevent the increased risk of DGF.^73,90^

#### ECD Kidneys

There is growing support for HMP for ECD kidneys and it has become standard practice in some centers. Again, a number of studies have compared HMP to SCS for ECD kidneys (Table 3). Jiao et al performed a meta-analysis of 7 studies involving 2374 HMP and 8716 ECD kidneys comparing HMP with SCS. There was a significant reduction in the OR of 0.59 (95% CI 0.54-0.66, *P* < 0.001) of DGF following HMP and improved graft survival at 1 y (OR 1.12, 95% CI 1.03-1.21, *P* = 0.005).^65^

In a subanalysis of the European RCT of 91 ECDs (182 kidneys), Gallinat et al^66^ demonstrated that HMP reduced the risk of DGF (OR 0.460, *P* = 0.047), lowered PNF rates by 9%, and improved graft survival at 1 y (92.3% versus 80.2%, *P* = 0.02) compared to SCS. This benefit in graft survival persisted at 3 y posttransplant.

From French registry data, Savoye et al^67^ found that the rate of DGF was significantly reduced in HMP kidneys (24%; n = 801) compared with SCS kidneys (38%; n = 3515) (OR 0.49 [0.40–0.60]). This result was also confirmed in a subanalysis of 66 pairs of ECD kidneys. HMP also reduced the risk of 1-y graft loss (OR 0.77 [0.60–0.99]).

In contrast in a US study, Basu et al^68^ found HMP had no significant effect on DGF (HMP 20.8% versus cold storage 25.8%). Kidney graft survival at 1 y was similar but at 3 y it was improved in the SCS kidneys. Six-year graft survival was 64.3% in HMP kidneys and 51.5% in SCS kidneys (*P* = 0.22). A possible explanation for the lack of effect was that the study included a high percentage of imported kidneys which were only placed on HMP after a significant period of CI. The CIT was also significantly longer in the HMP kidneys (28.9 h versus 24 h, *P* = 0.003).^68^

#### cDCD Kidneys

HMP with UW-MP solution is used for the majority of cDCD kidneys in Europe and in the United States. There have been a number of studies assessing the effects of HMP compared to SCS in cDCD kidneys (Table 3). In a recent meta-analysis, Tingle et al concluded from analysis of 7 studies including 772 DCD kidneys that HMP reduced the risk of DGF compared to SCS (RR 0.75 [0.64–0.87], *P* = 0.0002). There was strong evidence that HMP also improved graft survival at 1 and 3 y posttransplant.^69^ The systematic review and meta-analysis reported by Peng et al^62^ found that the risk of DGF in HMP DCD kidneys was significantly reduced (RR 0.73, 95% CI 0.61-0.88, *P* = 0.0010).

Using data from the European RCT comparing HMP and SCS in 82 pairs of DCD kidneys, Jochmans et al^70^ reported that the rate of DGF was reduced from 69.5% in the SCS group to 53.7% in the HMP group (OR 0.43, 95% CI 0.20-0.89, *P* = 0.025). However, 1-y graft survival was similar (93.9% versus 95.1%).

The PPART study reported by Watson et al^71^ from the United Kingdom included 45 pairs of kidneys from DCD donors. There was no significant difference in DGF (cold storage 56% versus HMP 58%). The main criticism of the trial was that kidneys were only placed on the HMP system (LifePort Kidney Transporter) on arrival at the recipient center after a period of SCS. Summers et al conducted a following RCT of pairs of kidneys from 51 DCD donors: kidneys underwent HMP (n = 51) for the entire preservation period and were compared to SCS kidneys (n = 51).^71^ DGF rates in the SCS group were 62.8% versus HMP 58.8% (*P* = 0.69). PNF in the SCS group was 5.9% and HMP 3.9% (*P* = 0.65). It is difficult to draw a conclusion from this study as the trial was underpowered and ended prematurely due to difficulty in recruiting. Of note, 53% of the DCD kidneys were from older donors.^72^ Other smaller studies have found significant reduction in the rate of DGF.^91^ Patel et al^73^ published the results of a cohort of kidneys undergoing HMP in the United Kingdom between 2007 and 2015. Rates of DGF were significantly lower than for SCS kidneys (34.2% versus 42.0%; *P* < 0.001). A marginal functional benefit was found at 1 y but there was no difference in graft survival.

#### uDCD Kidneys

Pieter Hoogland et al^8^ reported the outcome of the earliest series of uDCD kidney transplants (n = 135) from 1981 to 2009 in the Netherlands: the DGF rate was 61% and the PNF rate 22%. All kidneys were flushed in situ with HTK solution and the majority preserved by HMP with Belzer UW solution from 1985. Before that Euro-Collins solution was used for HMP.

In a series of uDCD kidneys in France from 2007 to 2010, Abboud et al^33^ reported a DGF rate of 95% and PNF rate of 5%. All kidneys were preserved by HMP. Patient and graft survival at 1 y were 98% and 91.4%, respectively. Sanni et al^92^ reported on a series of 100 DCD kidneys, of which 46% were uDCD. Kidneys were retrieved after rapid cooling in situ using HTK solution with 1.5 million units of streptokinase added in a preflush. All kidneys were then machine perfused with Belzer solution at 4 °C. The incidence of DGF was 62%, significantly higher than in a control group of 100 DBD kidneys (*P* = 0.0002).

### Hypothermic Oxygenated Machine Perfusion

Experimental studies suggest that even at hypothermic temperatures the addition of oxygen during perfusion can support ATP synthesis and prevent the deterioration of mitochondrial redox homeostasis to protect against preservation injury.^93^ This has been studied extensively in the liver.^94^ In the kidney, the addition of oxygen appears to be particularly beneficial when organs have been subjected to a period of WI injury.^95^ Buchs et al^95^ found that levels of ATP were restored in porcine kidneys after 30 min of WI injury. However, oxygenation had no added benefit in kidneys without injury.

In a recent experimental study, Kaminski et al^96^ monitored cortical tissue levels of ATP and oxygen during HMP and SCS. They found that in kidneys with WI injury, levels of ATP were higher during HMP and oxygen consumption increased compared to SCS.

It also appears that supplementing HMP with oxygen, recently termed hypothermic oxygenated machine perfusion (HOPE), for short durations can be used to resuscitate and condition organs.^97^ ATP can be replenished to reduce levels of oxidative stress and improve organ viability. Koetting et al^97^ demonstrated the advantage of adding oxygen during a 90-min period of hypothermic reconditioning to recover organs after ischemic injury with a 3-fold improvement in renal clearance of creatinine. In a rodent model, Kron et al^98^ found that 1 h of HOPE reduced cytokine release and resulted in less T-cell and macrophage activation. They also demonstrated improved function and less early fibrosis compared to untreated controls.

Darius et al^99^ advocated the use of a high concentration of oxygen during HMP and found that conditioning with oxygen at the beginning or for the entire 22 h of HMP was preferable to 2 h of HOPE at the end. The mitochondria were better preserved and levels of succinate, lactate, and flavin mononucleotide were significantly reduced compared to kidneys undergoing HMP without oxygen. The optimal concentration of oxygen administered during HMP is still debated. Although studies have shown benefit with the administration of high concentrations (100% and 95%), a concentration of 21% was also shown to reduce oxidative stress and improve the energy status.^100^

These preliminary research studies have led to the adaption and development of commercially available HMP systems that support oxygenation. LifePort, Kidney Assist, RM3, and WAVES either have membrane oxygenators incorporated into the circuit or they can be easily adapted with an external oxygenator added. There is also some evidence that oxygen can be simply bubbled into the perfusate during HMP to maintain an adequate partial pressure of oxygen, although lower than is achieved with an added oxygenator.^99^ A new system VitaSmart (Bridge to Life) is a multiorgan pump (liver and kidney) designed for use in the operating theater. The system relies on the simplicity of placing the kidney in a basin of cooled preservation solution on the back table in preparation for benching. The kidney is connected to the system via the renal artery and oxygenated preservation solution continually recirculated using a roller pump until ready for transplantation.

Oxygen can also be applied during SCS or HMP with the use of an extracellular hemoglobin called M101 isolated from the marine lugworm *Arenicola marina* (HEMO_2_life; Hemarina, Morlaix, France). M101 can release oxygen across a gradient over a range of temperatures. It also has antioxidant properties to protect against ischemia-reperfusion injury.^101^

#### ECD Kidneys

To reduce rates of DGF and improve outcome, there have been several recent studies examining the effect of HOPE on ECD kidneys (Table 3). In 2019, the Consortium for Organ Preservation in Europe (COPE) completed a RCT investigating HOPE after SCS versus SCS (COPE-POMP ISRCTN 63853508). The primary end point was 1-y graft survival and the results are awaited. In Germany, Meister et al^102^ demonstrated the feasibility of HOPE in a preliminary report of 2 pairs of ECD kidneys, one of each pair treated with 2–3 h of HOPE following SCS using the Kidney Assist Transporter and the other SCS. The same group is currently extending this series to include 15 ECD kidneys with a primary end point of dialysis within the first week of transplantation.^103^

In a recent safety study, Le Meur et al^104^ added the M101 oxygen carrier during HMP of ECD kidneys. A series of DBD kidneys undergoing SCS with added M101 was also included in the study. No adverse effects were noted and there was a small decrease in the rate of DGF.

#### cDCD Kidneys

The COPE consortium recently reported the results of an international double-blind multicenter trial comparing HMP with and without oxygenation using the Kidney Assist Transporter device in pairs of kidneys from >50 y DCD donors (COPE-COMPARE ISRCTN32967929; Table 3). The primary outcome measure was renal function at 12 mo posttransplant. There was no significant difference in graft function when comparing the 83 pairs that reached the primary end point (mean difference in estimated glomerular filtration rate [eGFR] 3.7 mL/min/1.73 m^2^, 95% CI 1.0-8.4; *P* = 0.12).^74^ The incidences of severe complications and episodes of acute rejection were lower in the oxygenated HMP group suggesting a reduction in the immunogenicity of the grafts.^105,106^ In a recent Italian series, 10 DCD kidneys were preserved by HOPE after normothermic regional perfusion (NRP). The rate of DGF was 30% with no reports of PNF.^107^

### Normothermic Machine Perfusion

Perfusing organs at sub- or near-normal temperatures has become the subject of much research over the last decade. Rather than suppressing metabolism, the conditions are designed to support aerobic metabolism and restore cellular function. This has a number of potential advantages over SCS and standard hypothermic techniques. Injury caused by CI during SCS can be avoided or minimized, it may allow the upregulation of repair mechanisms and also provides the opportunity to make a functional assessment of the kidney. The disadvantage of normothermic machine perfusion (NMP) is that the restoration of cellular function also insights the upregulation of inflammatory mediators that can potentially cause harm.^108^ Experimental studies have explored techniques using red blood cell–based solutions to support oxygen delivery^109^ or artificial oxygen carriers such as Hemopure (hemoglobin glutamer-250 [bovine]; HBOC-201, Hemoglobin Oxygen Therapeutics LLC)^110^ and pyridoxylated bovine hemoglobin reported by Brasile et al.^111^ Brasile et al^111^ conducted a significant amount of research from the 1990s using an exsanguinous metabolic support medium (Breonics) made up of a highly enriched tissue culture-like medium containing essential and nonessential amino acids, lipids, and carbohydrates supplemented with bovine hemoglobin to perfuse canine, porcine, and nontransplanted human kidneys.^112^ They demonstrated that perfusing kidneys at 32 °C for short periods could protect against ischemic injury and that more prolonged periods of perfusion (24 h) could promote recovery and repair.^111,112^ The addition of growth factor or mesenchymal stromal cells to the kidneys during perfusion could further promote recovery.^113,114^

Nicholson and Hosgood used more physiological conditions by perfusing porcine and human kidneys with a red blood cell–based solution at 35–36 °C.^109,115^ NMP was performed for a short 1–2 h period after hypothermic preservation. Experimental evidence showed that ATP could be replenished and protective mechanisms such as heat shock protein 70 were upregulated.^115^ More recently using a similar protocol Hameed et al^116^ examined the transcriptional changes in gene expression in pairs of nontransplanted human kidneys. A 1-h period of NMP-activated protective stress responses and promoted cell survival and proliferation.

Kaths et al^117^ found that more prolonged periods of NMP (8–16 h) were necessary to recover function after WI and CI injury. They used a red blood cell–based solution mixed with STEEN solution. STEEN solution was originally formulated for the lung and contains a high concentration of albumin and dextran to create a high osmotic pressure. For NMP of the kidney, it is necessary to dilute it with a crystalloid solution to prevent cellular damage and diffuse vacuolation of the tubular cells. Weissenbacher et al^118,119^ also advocated the use of prolonged periods of NMP and demonstrated that by recirculating the urine the kidney could maintain a more stable environment.

Gallinat et al^120^ also used a preservation medium based on STEEN solution to gradually rewarm kidneys after WI and CI injury. The protocol involved a 90-min controlled phase of rewarming before NMP at 35 °C using STEEN solution. The experimental evidence demonstrated the adequate delivery of oxygen without the addition of an oxygen carrier.^121^ Gradual rewarming protected against mitochondrial and cellular injury by reducing levels of damage-associated molecular patterns (toll-like receptor 4 and high mobility group box 1 protein) during reperfusion.^122^ Furthermore, it could more efficiently optimize ATP and oxygen consumption levels during reperfusion compared to the immediate transition from 4 °C.^122^

#### Assessment

The increasing interest in NMP for kidney preservation is also focused on its use as a device to assess the viability or quality of the kidney. The higher level of cellular function compared to HMP allows a functional assessment in addition to the cellular biomarkers used during HMP (Table 4).^123^ More recently, the application of metabolomic and transcriptional analysis has been investigated and may help to reveal future biomarkers (Table 4).

#### Technologies

At present, the Kidney Assist device made by Organ Assist based in the Netherlands is the only commercially available CE-marked NMP system for the kidney. Other reported systems are in-house adaptations of current cardiopulmonary bypass technology or custom-made perfusion devices. The UK-based company OrganOx has designed a prototype portable system for maintaining a kidney under NMP conditions for prolonged periods but it is not yet available on the market.

#### DBD Kidneys

NMP has not been reported for the preservation of standard DBD kidneys. However, the Toronto group is conducting a pilot study (n = 25) investigating the effects of 1–10 h of normothermic ex vivo kidney perfusion with a blood-based solution in DBD and DCD kidneys.

#### ECD Kidneys

NMP using a red blood cell–based perfusate and adapted cardiopulmonary bypass system was first introduced into clinical practice in December 2010 in the United Kingdom.^109^ A kidney from an ECD rejected by 5 other centers in the United Kingdom was transplanted successfully after 1-h NMP. The recipient had slow graft function for 1 mo but remained dialysis-free.

In 2013, the feasibility and safety of NMP was established in a series of 18 ECD kidneys.^75^ The DGF rate was 11% compared to 36.2% in a matched series of historical control kidneys that had SCS only; 1-y graft survival and patient survival were similar (Table 3). Hosgood and Nicholson^124^ also reported that it was feasible and safe to perform NMP then place an ECD kidney back on ice for a further 5 h until transplantation.

Most recently Minor et al used the Kidney Assist device and the controlled rewarming approach with STEEN solution to perfuse an ECD kidney. After 90 min of controlled rewarming, the kidney was successfully transplanted with immediate graft function.^125^

In the Netherlands, a pilot study has recently been completed that assessed the effects of 2–6 h of NMP using the Kidney Assist device in ECD kidneys.^126^

#### cDCD Kidneys

Nicholson and Hosgood have reported several cases of DCD kidneys undergoing 1-h NMP. In each of the cases, NMP was used to salvage the kidneys after they were inadequately flushed during retrieval.^127,128^ In 1 case, the kidney was retrieved after NRP. In both cases, the kidneys performed well during NMP and were transplanted successfully.

With the promising results from the series of ECD kidneys, the NMP technology is being assessed in a RCT of DCD kidneys.^129^ Kidneys are randomized to receive either SCS or NMP in a 1:1 ratio. The trial is due to report in 2021 (ISRCTN15821205).

There is 1 report of using NMP technology to avoid any exposure to CI. He et al^130^ adapted the Kidney Assist device to perfuse a DCD kidney by cannulating the infrarenal abdominal aorta and suprarenal inferior renal cava and transferring to the Kidney Assist device while maintaining circulation. The kidney was perfused for 110 min with a red blood cell–based solution before implantation into the recipient. Circulation was maintained throughout and the recipient had immediate graft function.

### Normothermic Regional Perfusion

Traditionally, organs from deceased and DCD donors are rapidly flushed in situ with cold preservation solution to reduce the temperature and lower the metabolism as quickly as possible to prevent cellular injury. NRP involves the restoration of circulation with the donor’s own blood using extracorporeal membrane oxygenation technology after confirmation of circulatory arrest. NRP can be carried out over a range of temperatures, subnormothermic (4–22 °C) or normothermic (35–37 °C). In the United Kingdom, the donor is heparinized after cardiac death to prevent clotting, but heparinization is permitted before cardiac death in some European protocols. Circulation is restored for a variable period of time (1–6 h). NRP is thought to condition the organs by upregulating adenosine receptors which may protect against preservation injury. In a recent porcine study, NRP was found to enhance the expression of protective mechanisms (erythropoietin, heme-oxygenase-1, glucose transport 1, and vascular endothelial growth factor) via the hypoxia-inducible factor 1α signaling pathway.^131^ They found levels of aspartate aminotransferase decreased over 4 h of NRP, which suggested a reduction in injury and a possible reconditioning effect. NRP was difficult to maintain after 4 h and an increase in inflammatory markers with high levels of monocyte chemoattractant protein-1, interleukin 1β and significant macrophage infiltration, together with increased platelet activation and expression of thrombomodulin, suggest that it may have some detrimental effects.^131^

Donor Assist (Organ Assist) is the one commercially available dedicated NRP system. Other systems are adapted extracorporeal membrane oxygenation or cardiac bypass systems (Maquet, Medtronic).

#### cDCD Kidneys

In situ cooling has been the standard method of cooling before organ retrieval for all deceased donors. However, there is growing interest in NRP for DCD donors. Early series of NRP in cDCDs demonstrated that its application could increase the number of available organs with good outcomes. DGF rates were reported at 8.3% and 11%.^132,133^ In comparison to in situ perfusion or total body cooling the incidence of DGF and PNF was lower in the NRP group (Table 3).^134^

More recently, in the first series from the United Kingdom, the outcomes of 14 kidneys from 8 cDCD donors were reported: 1 pair of kidney was not used due to a high Remuzzi score; 1 pair had PNF and were removed 5 d posttransplant; and 2 recipients had DGF (18%).^135^ In the second study from the United Kingdom, Oniscu et al^136^ reported on 21 NRP retrievals from 3 centers in the United Kingdom. Thirty-two kidneys were transplanted with a DGF rate of 40%. Miñambres et al^23^ reported the first Spanish series of 27 NRP donors from which 37 kidneys were transplanted and reported a DGF rate of 27%. They compared the results to a series of 51 DBD donors and found no statistically significant difference in graft survival 18 mo posttransplant (91.8% versus 97.2%).^23^ In an early series from the United States, between 2000 and 2013, the rate of DGF in 48 kidneys retrieved from 37 cDCD donors was 31%.^38^ Several other studies have found comparable outcomes with DBD kidneys.^23,27,28,76,137^ The largest study from France included 92 kidneys from NRP donors and 5176 DBD donors and reported significantly lower levels of DGF (9% versus 19%, *P* < 0.05).^76^ Kidneys in the NRP cohort all underwent HMP following NRP.

#### uDCD Kidneys

Some European countries advocate the application of NRP in their uDCD programs to reduce the risk of PNF and increase utility of organ donors.^27^

Reznik et al^138^ reported the application of subnormothermic NRP (27–32 °C) in uDCD donors using leukocyte-depleted blood. Forty-four kidneys were transplanted from 22 donors. The DGF rate was 52.3% and there were no incidences of PNF. Graft survival at 1 y was 95.5%.

Results from large studies in Spain and France are encouraging. In Madrid, 237 kidneys were transplanted after NRP and compared to 237 DBD kidneys. The rate of DGF was 73.4% versus 46.4% (*P* < 0.01) and graft and patient survival similar at 10 y (82.1% versus 80.5%, *P* = 0.623; 86.2% versus 87.6%, *P* = 0.454). PNF rate was 6.8% versus 4.2% in the DBD kidneys.^28^ In another study including 517 kidneys from 445 uDCD donors across different centers in Spain, the incidence of DGF was 76%, PNF rate was 10%, and graft survival at 1 y was 87%.^137^ A proportion of the donors in this study were >60 years of age and therefore the risk of PNF enhanced. Overall, NRP strategies were preferable to in situ cooling.

In a study from France including 499 kidneys from 414 uDCD donors across 15 different transplant centers from 2007 to 2014, Antoine et al^76^ found that the risk of PNF and graft loss was significantly higher if NRP was not used (OR 2.6, CI 1.5-4.6). In a smaller study from Portugal of 44 kidneys from 40 uDCD donors, the incidence of DGF was 68% and PNF 9%.^139^

## CONCLUSIONS

**FIGURE 1.**
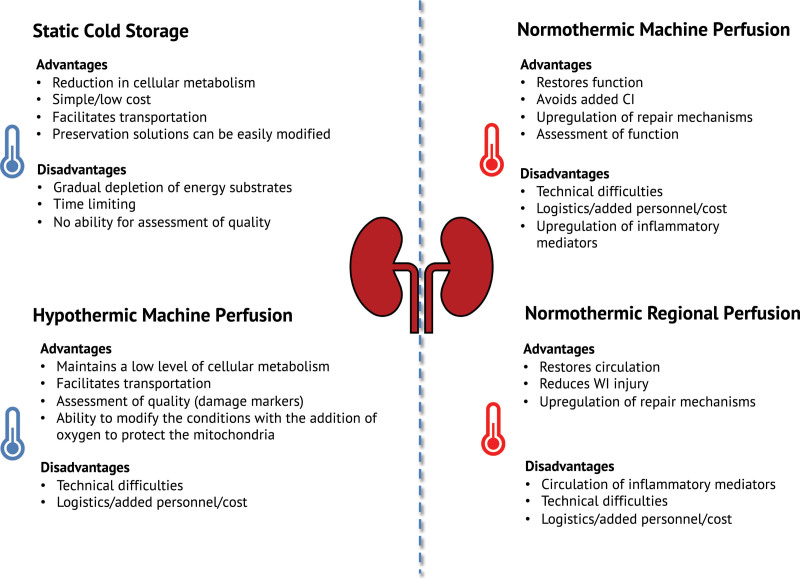
Advantages and disadvantages of different types of kidney preservation. CI, cold ischemia; WI, warm ischemia.

This review provides an overview of the different techniques of kidney preservation and evidence for their application in clinical transplantation for each type of organ donor. Each technique has advantages and disadvantages listed in Figure 1. SCS is a robust method of preservation and results suggest that for living donor and SCD donor kidneys it is a satisfactory method of preservation with no clear advantage of 1 type of preservation solution over another. Nonetheless, UW solution remains the gold standard and most widely used in deceased donor transplantation. There has been little emphasis on the development of new SCS preservation solutions since the 1990s and in recent times few studies have assessed the effects of different solutions, particularly in DCD kidneys. Much of the research has focused on comparing HMP with SCS techniques, with many studies showing reduced risk of DGF.

Since 2016, HMP has become standard practice in the Netherlands for all deceased donor kidneys including SCDs. HMP has received significant support from industry, with a number of commercially available systems on the market. Nonetheless, HMP is not supported by all studies. Furthermore, there is limited evidence for the benefits of HMP on graft survival compared to SCS techniques.

Active oxygenated HMP is an encouraging technique of preservation and has shown significant advantages in liver transplantation. In the kidney, the results of the COPE consortium RCT in DCD donors >50 y demonstrated the safety and feasibility of added oxygen which also may transfer some health-economic benefit of the technique. The results from the COPE led in ECD kidneys are eagerly awaited. The novel application of the oxygen carrier M101 to the preservation solution during SCS or HMP is also a promising new strategy to reduce the effects of CI and warrants further assessment.

NMP is also a promising new technique of preservation that offers more than a simple means of preservation. The ability to assess function, reverse the effects of ischemic injury and its potential use as platform for the delivery of therapeutic agents have significant advantages (Figure 1). However, at present, there is a limited amount of evidence for clinical benefits compared to hypothermic techniques. The utilization of NMP is increasing, with results from small case series of ECD kidneys in the Netherlands and DBD/DCD kidneys in Canada expected this year. NMP can be carried out over a range of temperatures, for different durations and using different compositions of perfusate.^140^ Further experimental work is required to optimize NMP conditions and to investigate the mechanistic actions before more widespread use in clinical practice.

The results from the application of NRP are also encouraging particularly for increasing the utility of uDCD donors. Although the rates of DGF and PNF are high, graft survival is similar to other deceased donor kidneys. Nonetheless, the rate of organ utilization could be improved and there is no firm evidence of the benefits compared to standard cold in situ organ retrieval.

At present, the number of different preservation techniques is growing and, in the future, there are likely to be a range of different modes of preservation available in kidney transplantation tailored for specific types of donor kidney. Logistics and costs are factors that remain challenging and the application of these technologies may not be available to all (Figure 1). There is a lack of well-designed RCTs comparing different techniques of preservation as highlighted in Table 4. More RCTs are needed to determine the best mode of preservation for specific types of donor kidney, with particular focus on improving long-term graft function and survival. The development of reliable biomarkers to assessment viability and predict outcome is underway, with interest in exosomes and nanoparticles. The application of transcriptional and metabolomic approaches, particularly with the use of NMP, will allow the refinement of preservation techniques and improve outcomes.
